# [Corrigendum] Isolinderalactone enhances the inhibition of SOCS3 on STAT3 activity by decreasing miR-30c in breast cancer

**DOI:** 10.3892/or.2025.9041

**Published:** 2025-12-22

**Authors:** Meng-Chi Yen, Ying-Chu Shih, Ya-Ling Hsu, En-Shyh Lin, Yi-Shiuan Lin, Eing-Mei Tsai, Ya-Wen Ho, Ming-Feng Hou, Po-Lin Kuo

Oncol Rep 35: 1356–1364, 2016; DOI: 10.3892/or.2015.4503

Subsequently to the publication of the above paper, an interested reader drew to the authors' attention that, regarding the western blot data shown in Fig. 4 on p. 1361, the lower of the control GAPDH blots in Fig. 4A and B appeared to be essentially the same (albeit the blots were more exposed in Fig. 4B), even though the experimental conditions in the two figure parts were reported to be different.

The authors were able to re-examine their original data files, and realized that the western blot image for Fig. 4B had inadverently been selected incorrectly. The revised version of Fig. 4, now containing the correct data for the lower GAPDH blots in Fig. 4B, is shown below. Note that the correction made to this figure does not affect the overall conclusions reported in the paper. The authors are grateful to the Editor of *Oncology Reports* for allowing them the opportunity to publish this Corrigendum, and apologize to the readership for any inconvenience caused.

## Figures and Tables

**Figure 4.**
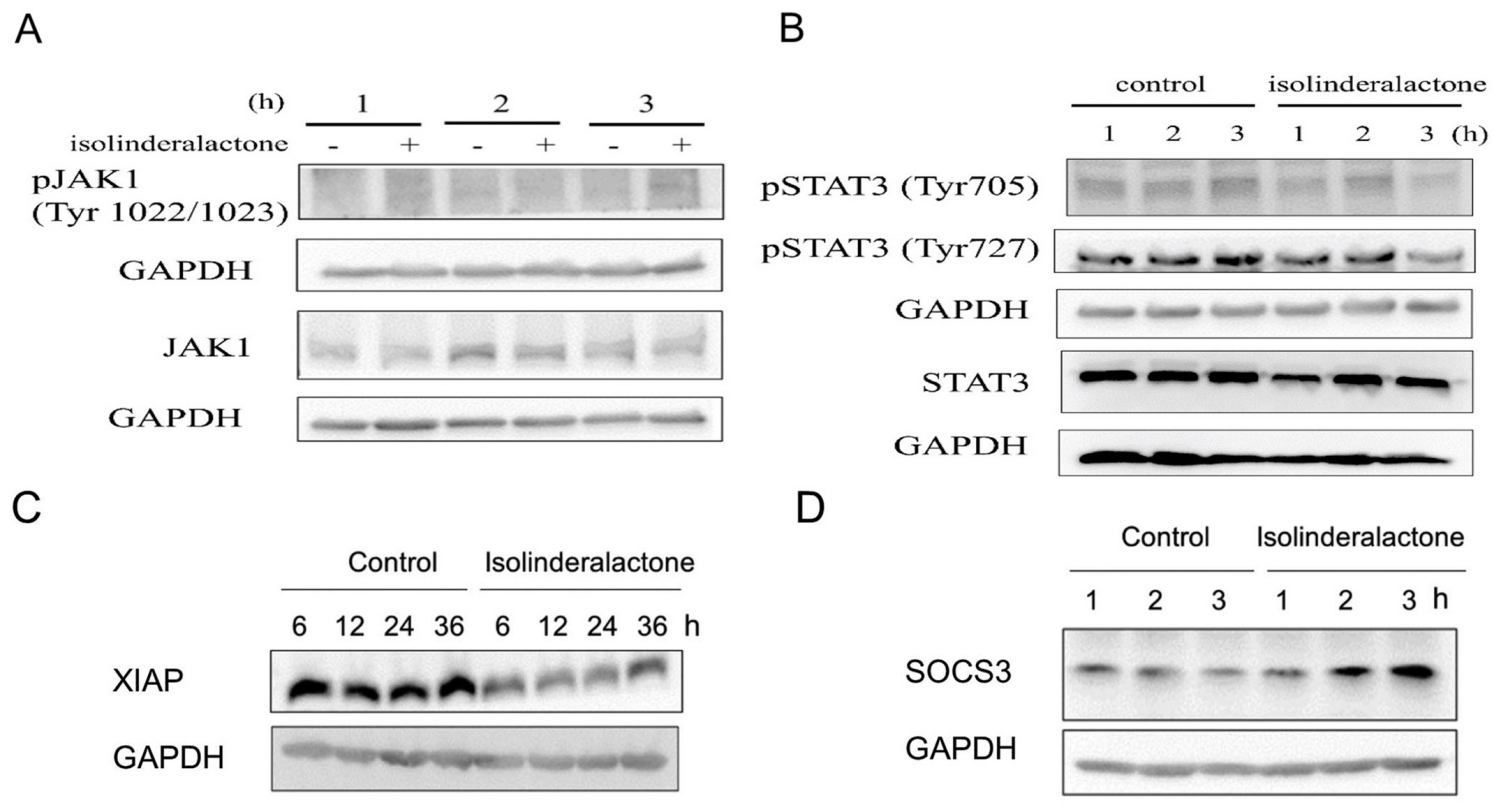
Isolinderalactone treatment suppresses phosphorylation of STAT3 through SOCS3-dependent pathway. MDA-MB-231 cells were treated with vehicle or 20 µM isolinderalactone at different time points. The expression was analyzed by western blotting. (A) The expression level of JAK1 and phosphorylated-JAK1. (B) The expression level of STAT3 and phosphorylated-STAT3. (C) The expression of XIAP. (D) The expression of SOCS3. Data are representative of at least three independent experiments.

